# Insights into the survival impact of delayed immunochemotherapy in newly diagnosed diffuse large B-cell lymphoma: results from a nationwide registry study

**DOI:** 10.1186/s12885-026-17024-9

**Published:** 2026-09-26

**Authors:** Susanne von Kroge, Nils Janis Steinhaus, Carsten Bokemeyer, Fabian Tetzlaff, Hanna Walk, Henrik Kusche, LYSED study group, Sabrina Klotz, Nadia Obi, Olaf v.d. Knesebeck, Mareile Beernink, Christopher Gundler, Frank Ückert, Katharina Schulze, Jobst Augustin, Regina Semmelhaack, Anastasia Sassenberg-Gummert, Frederik Peters

**Affiliations:** 1 Department of Oncology, Hematology and Bone Marrow Transplantation With Section Pneumology, University Cancer Center Hamburg, University Medical Center Hamburg-Eppendorf, Martinistraße 52, Hamburg, 20246 Germany; 2 Division of Social Determinants of Health, Department of Epidemiology and Health Monitoring, Robert Koch Institute, Nordufer 20, Berlin, 13353 Germany; 3 Hamburg Cancer Registry, Ministry of Science, Research and Equality, Free and Hanseatic City of Hamburg, Süderstraße 30, Hamburg, 20097 Germany

**Keywords:** Diffuse large B-cell lymphoma, Diagnosis-to-treatment interval, Treatment delay, Survival

## Abstract

**Background:**

The prognostic impact of the diagnosis-to-treatment interval (DTI) in diffuse large B-cell lymphoma (DLBCL) remains controversial. We investigated the association between DTI and three-year all-cause mortality in a nationwide cohort.

**Methods:**

We conducted a retrospective population-based cohort study using harmonised German cancer registry data (2020–2023), with vital status follow-up until December 2024. Patients with a first primary DLBCL diagnosis were included. Kaplan–Meier analyses and Cox regression assessed DTI in relation to survival, unadjusted and adjusted for patient, tumor, and structural characteristics. Missing International Prognostic Index (IPI) data formed a separate category, with complete-case and imputation sensitivity analyses.

**Results:**

Of 16,443 patients, 10,872 initiated systemic therapy within 56 days (eight weeks) and entered the primary analysis. Median age was 71 years, 44.2% were female, and median follow-up was 1.5 years. Three-year overall survival ranged from ~ 63% to 75% across the five treatment-week groups. Compared with week 1, later initiation was associated with progressively lower hazard of death (week 3: HR 0.72, 95% CI 0.64–0.81; week 4: HR 0.63, 0.55–0.72; weeks 5–8: HR 0.62, 0.54–0.70). Lower-risk IPI predicted better survival (low risk: HR 0.24, 0.17–0.34 vs high risk). Later initiation was predicted by low-risk IPI (+ 7.89 days), older age (+ 0.62 days/SD), and surviving the first year (+ 3.26 days).

**Conclusion:**

Early treatment in DLBCL is primarily driven by adverse disease characteristics. A residual association between longer DTI and improved survival persisted after IPI adjustment, suggesting DTI may be an independent prognostic factor in less aggressive disease.

**Supplementary Information:**

The online version contains supplementary material available at https://doi.org/10.1186/s12885-026-17024-9.

## Introduction

Diffuse large B-cell lymphoma (DLBCL) is the most common aggressive B-cell lymphoma and a rapidly progressing malignancy that requires prompt treatment. Despite current first-line treatment approaches combining CD20-targeted antibodies, and, in some cases CD79b-targeting antibody–drug-conjugates with conventional anthracycline-based chemotherapy, approximately 30% of patients develop refractory or relapsed disease [1]. In younger patients first-line treatment decisions are primarily guided by the International Prognostic Index (IPI), which incorporates age, number of extranodal sites, lactate dehydrogenase levels, and disease stage [2].

Over the past two decades, evidence on the prognostic impact of diagnosis-to-treatment interval (DTI) in cancer has been inconsistent across different cancer entities [3]. A systematic review by Neal et al. (2015) highlighted only moderate consensus regarding associations between diagnostic intervals and clinical outcomes, with studies reporting shorter intervals to be associated with better, worse, or no differences in outcomes [3]. For newly diagnosed DLBCL, findings on the prognostic impact of DTI remain contradictory. While most evidence points towards an association between shorter DTI and high-risk disease characteristics, with subsequent decreased survival [4–10], other studies report the opposite association [11] or no association with survival at all [12]. Moreover, the association between a shorter DTI and high-risk disease characteristics is discussed in the context of a selection bias, leading to an underrepresentation of DLBCL patients with unfavorable clinical characteristics in clinical trials [7, 13].

To date, it remains unclear whether there are prognostically relevant delays in treatment initiation among DLBCL patients within a universal healthcare system. Therefore, we aimed to investigate the prognostic impact of DTI on long-term outcomes in newly diagnosed DLBCL patients by conducting a large, nationwide, cancer registry-based cohort study.

## Materials and methods

### Study design and data sources

We conducted a retrospective population-based cohort study using a validated, harmonised pooled clinical cancer registry dataset from the Robert Koch Institute, covering Germany (~ 83 million inhabitants) for 2020–2023, with vital-status follow-up until 31 December 2024 [14]. German registry data have been validated for assessing DLBCL outcomes [15–17]. Data were available for 16 federal states (NUTS-1) and 401 districts (NUTS-3; 2020 boundaries). District-level data on urbanity and premature mortality were taken from the Federal Institute for Research on Building, Urban Affairs and Spatial Development [18], deprivation from the German Index of Socioeconomic Deprivation (GISD) [19], certified-centre locations from the German Cancer Society (www.oncomap.de, accessed 01/04/2026), and age-/sex-specific life-table death probabilities from the Human Mortality Database [20].

### Patients

We included all patients with primary DLBCL (ICD-10-GM C83.3, excluding ICD-O-3 topography C70–C72) diagnosed between 1 January 2020 and 31 December 2023. We excluded patients aged < 19 or > 99 years, with unknown sex, follow-up < 56 days, or unknown district/residence in a state with incomplete coverage (North Rhine-Westphalia). Only the first tumour was retained.

### Study variables and exposure

Patient-level variables were federal state, calendar year, age, sex, and the International Prognostic Index (IPI). District-level variables were settlement structure (rural sparsely populated, rural with agglomeration tendencies, urban, large city), presence of a certified lymphoma centre, premature mortality (deaths/1,000 residents before age 70; comorbidity proxy), and the continuous GISD score (0–1; higher = more deprived). The exposure, days to treatment initiation (DTI), was the number of days from diagnosis to first systemic therapy (earliest date if multiple); implausible values (negative, > 365, or missing) were treated as no therapy. The primary exposure was treatment within 56 days (eight weeks), categorised by week: 1 (days 1–7), 2 (8–14), 3 (15–21), 4 (22–28), and 5–8 (29–56). The cutoff of 56 days was chosen a priori because DLBCL is generally managed with prompt initiation of treatment and prior work suggested that patients with DTI exceeding 8 weeks represent a clinically distinct subgroup with different prognostic mechanisms [11].

### Outcomes

The primary outcome was all-cause mortality between 56 days and three years after diagnosis. Patients were followed from diagnosis until the earliest of death, 31 December 2024 (administrative end of follow-up), or 1,095 days; survivors were censored at that point. The secondary outcome was determinants of DTI.

### Statistical analysis

All analyses were performed in R (version 4.3.2). The significance level was set at α = 0.05. Categorical variables are reported as counts/proportions, continuous variables as medians (IQR), and group differences as standardized mean differences. Unadjusted survival by week of initiation was estimated by Kaplan–Meier methods with log-rank tests. Two multivariable models were fitted: (I) linear regression for factors affecting DTI (0–56 days) among patients treated within 56 days adjusting for sex, age at diagnosis, settlement type, death within 365 days of diagnosis, IPI, certified centre in district of residence, premature mortality in district of residence and GISD score in district of residence; and (II) Cox proportional-hazards models for the association between week of initiation and overall survival adjusting for sex, age at diagnosis, settlement type, IPI, certified centre in district of residence, premature mortality in district of residence and GISD score in district of residence. Model assumptions were assessed by visual inspection of the outcome distribution and residual diagnostic plots. All models included federal state and calendar year as fixed effects, with cluster-robust standard errors at district level. Unknown IPI was kept as a separate category; in model I, death between 56 and 365 days served as a frailty proxy. Fit was assessed with Harrell’s C-index and AIC.

## Results

### Study cohort

**Fig. 1:**
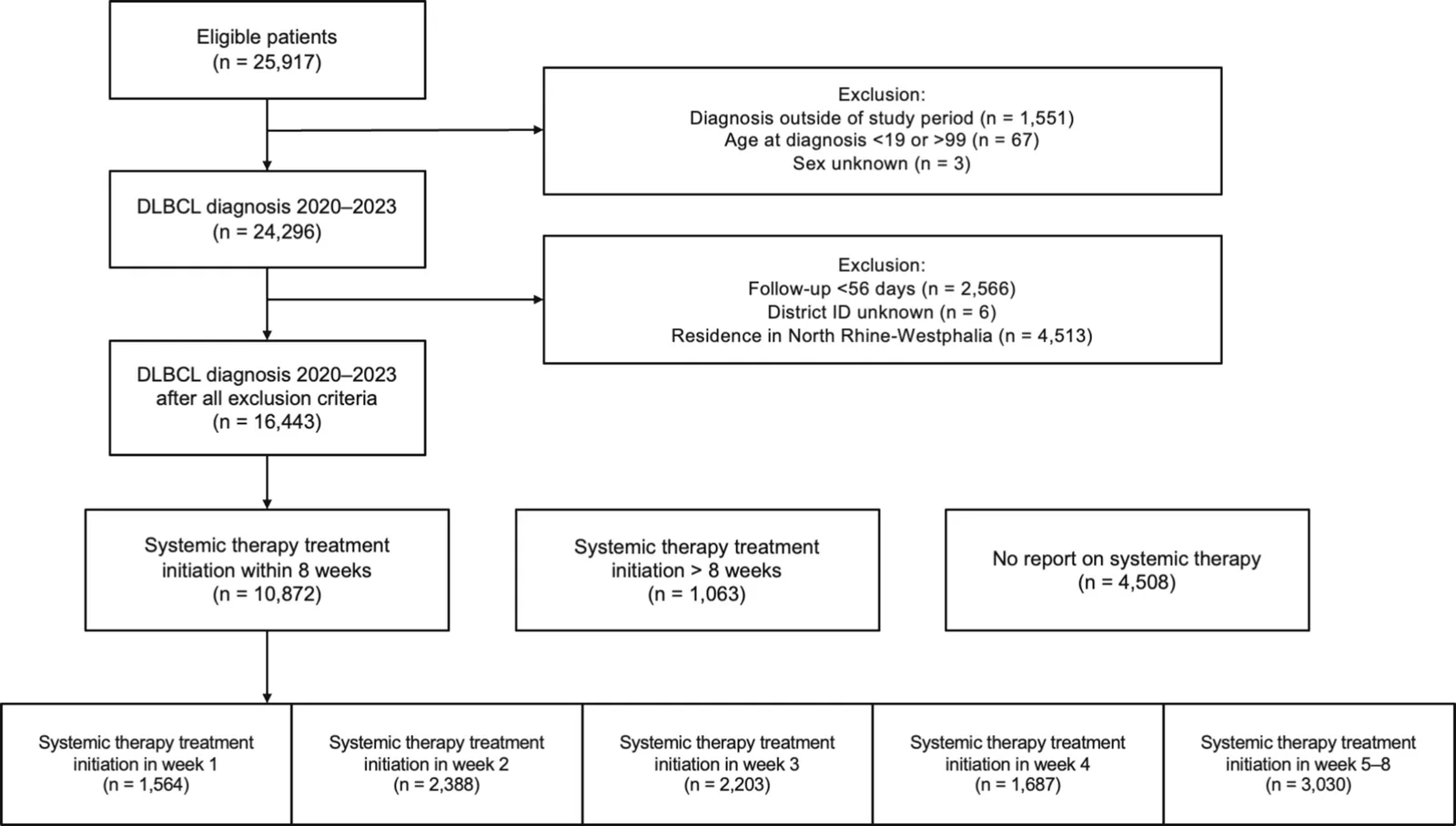
Flow chart of the study cohort

Of 16,443 DLBCL patients, 11,935 received a systemic therapy, of which10,872 patients-initiated therapy within eight weeks forming the final study cohort (Fig. 1). A separate analysis of those excluded from the analysis revealed that patient without report on systemic therapy (*n* = 4,508) were much older (age group 80 + : 37% vs. 26%, *p* < 0.001), died more often within three years after diagnosis (29% vs. 25%, *p* < 0.001), had less often a certified centre in their district (33% vs. 42%, *p* < 0.001) and also had more often no report in IPI (missing 95% vs. 73%, *p* < 0.001) than those with treatment initiation within 56 days (Table S1). The remaining 1,063 patients with treatment initiation later than eight weeks were more similar to those within eight weeks (Table S1). Mean age of the final study cohort was 69 years and 44.2% were female with a median follow-up time of 1.5 years (Table 1). A total of 1,564 (14.4%) patients initiated systemic therapy in week 1, 2,388 (22.0%) in week 2, 2,203 (20.3%) in week 3, 1,687 (15.5%) in week 4, and 3,030 (27.9%) in weeks 5–8. IPI data were available for 28% of patients in the analysis cohort; among those, the distribution across risk groups was: high risk 6.4%, high-intermediate risk 7.7%, low-intermediate risk 6.6%, and low risk 6.7% whereby the proportion of unknown IPI increased with time of treatment initiation.

**Table 1:** Baseline characteristics of the study cohort stratified by time to initiation of systemic therapy (unknown IPI score coded as a separate category; patients without systemic therapy initiation within eight weeks excluded)

Variables	Overall (*N* = 10,872)	1 week (*N* = 1,564)	2 weeks (*N* = 2,388)	3 weeks (*N* = 2,203)	4 weeks (*N* = 1,687)	5–8 weeks (*N* = 3,030)	SMD
Year of diagnosis, n (%)							0.09
2020	2,906 (26.7)	451 (28.8)	681 (28.5)	600 (27.2)	439 (26.0)	735 (24.3)	
2021	2,909 (26.8)	437 (27.9)	612 (25.6)	593 (26.9)	444 (26.3)	823 (27.2)	
2022	2,762 (25.4)	357 (22.8)	637 (26.7)	532 (24.1)	462 (27.4)	774 (25.5)	
2023	2,295 (21.1)	319 (20.4)	458 (19.2)	478 (21.7)	342 (20.3)	698 (23.0)	
Age in years, mean (SD)	68.98 (13.95)	67.71 (14.62)	69.44 (13.59)	69.39 (13.88)	68.78 (13.74)	69.10 (14.01)	0.06
Age group, n (%)							0.08
18–39, n (%)	469 (4.3)	87 (5.6)	86 (3.6)	94 (4.3)	68 (4.0)	134 (4.4)	
40–49, n (%)	571 (5.3)	100 (6.4)	110 (4.6)	105 (4.8)	96 (5.7)	160 (5.3)	
50–59, n (%)	1,353 (12.4)	195 (12.5)	304 (12.7)	272 (12.3)	216 (12.8)	366 (12.1)	
60–69, n (%)	2,467 (22.7)	357 (22.8)	563 (23.6)	470 (21.3)	385 (22.8)	692 (22.8)	
70–79, n (%)	3,160 (29.1)	456 (29.2)	679 (28.4)	658 (29.9)	501 (29.7)	866 (28.6)	
80 +, n (%)	2,852 (26.2)	369 (23.6)	646 (27.1)	604 (27.4)	421 (25.0)	812 (26.8)	
Female sex, n (%)	4,810 (44.2)	701 (44.8)	1,073 (44.9)	967 (43.9)	731 (43.3)	1,338 (44.2)	0.02
IPI, n (%)							0.20
High risk	701 (6.4)	172 (11.0)	209 (8.8)	119 (5.4)	85 (5.0)	116 (3.8)	
Low risk	731 (6.7)	73 (4.7)	109 (4.6)	147 (6.7)	140 (8.3)	262 (8.6)	
Low-intermediate risk	715 (6.6)	90 (5.8)	153 (6.4)	143 (6.5)	119 (7.1)	210 (6.9)	
High-intermediate risk	840 (7.7)	173 (11.1)	215 (9.0)	171 (7.8)	115 (6.8)	166 (5.5)	
Unknown	7,885 (72.5)	1,056 (67.5)	1,702 (71.3)	1,623 (73.7)	1,228 (72.8)	2,276 (75.1)	
Deaths up to three years, n (%)	2,703 (24.9)	494 (31.6)	711 (29.8)	530 (24.1)	353 (20.9)	615 (20.3)	0.15
Follow-up time in years, median (IQR)	1.54 [0.71, 2.63]	1.43 [0.66, 2.63]	1.46 [0.66, 2.54]	1.54 [0.74, 2.71]	1.58 [0.80, 2.71]	1.63 [0.80, 2.63]	0.06
Spatial type, n (%)							0.06
Rural sparsely	2,180 (20.1)	301 (19.2)	483 (20.2)	444 (20.2)	326 (19.3)	626 (20.7)	
Rural densely	2,293 (21.1)	312 (19.9)	497 (20.8)	454 (20.6)	366 (21.7)	664 (21.9)	
Urban	3,494 (32.1)	532 (34.0)	748 (31.3)	672 (30.5)	551 (32.7)	991 (32.7)	
Larger city	2,905 (26.7)	419 (26.8)	660 (27.6)	633 (28.7)	444 (26.3)	749 (24.7)	
Certified hospital center, n (%)	4,536 (41.7)	644 (41.2)	1,018 (42.6)	976 (44.3)	688 (40.8)	1,210 (39.9)	0.04
GISD score, median (IQR)	0.50 [0.39, 0.62]	0.48 [0.36, 0.60]	0.50 [0.38, 0.61]	0.50 [0.39, 0.62]	0.51 [0.40, 0.62]	0.52 [0.41, 0.62]	0.10
District level deaths before age 70, per 1,000 persons, median (Q1, Q3)	2.86 (2.51, 3.63)	2.78 (2.43, 3.44)	2.85 (2.51, 3.60)	2.87 (2.51, 3.65)	2.88 (2.51, 3.71)	2.91 (2.55, 3.68)	0.08

Values ≥ 0.10 were interpreted as non-negligible differences

*Abbreviations*: *IPI* International Prognostic Index, *IQR* Interquartile range, *Q1* First quartile, *Q3* Third quartile, *SD* Standard deviation, *SMD* Standardized Mean Difference

Patients who started treatment later within eight weeks did not differ markedly from those starting earlier except with respect to a more favourable IPI risk score (SMD = 0.20), and better survival during the follow-up period (SMD = 0.15). Furthermore, socioeconomic deprivation (GISD score) showed a difference across treatment-week groups (SMD = 0.10), with patients treated later tending to reside in more deprived districts.

### Predictors of study exposure

For the 10,872 that initiated systemic therapy within eight weeks after diagnosis, a later initiation of treatment was associated with surviving the first year after diagnosis (DTI + 3.26 days, 95% CI 2.54–3.99, *p* < 0.001) and older age (DTI + 0.62 days per standard deviation, 95% CI 0.39–0.86, *p* < 0.001). No relevant association was observed for socioeconomic deprivation or district-specific premature mortality) (Figure S1). The main predictor of a later initiation of systemic therapy was low risk IPI (DTI + 7.89 days, 95% CI 6.50–9.29; *p* < 0.001).

### Unadjusted overall survival

**Fig. 2:**
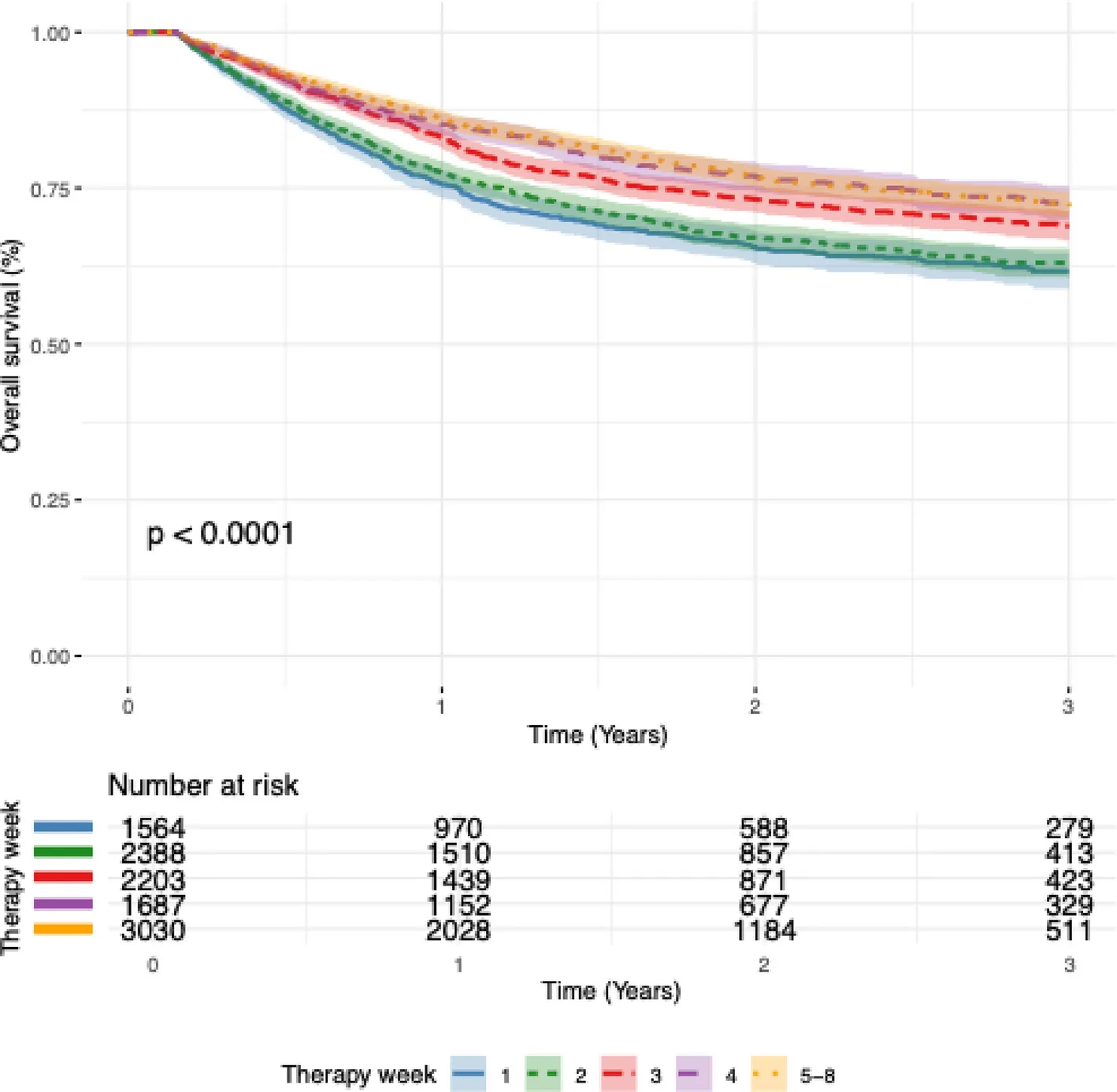
Unadjusted three-year overall survival with 95% confidence intervals in patients with DLBCL stratified by time-to-systemic-treatment (weeks)

OS after three years of follow-up ranged from approximately 63% to 75% and differed significantly across treatment-week groups (*p* < 0.001; Fig. 2). Survival was lowest among patients treated in week one and week two and markedly higher for those with treatment initiation at weeks three, four, and five to eight. Patients treated in week four and patients treated in weeks five to eight exhibited similar survival.

### Adjusted multivariate Cox regression

**Fig. 3:**
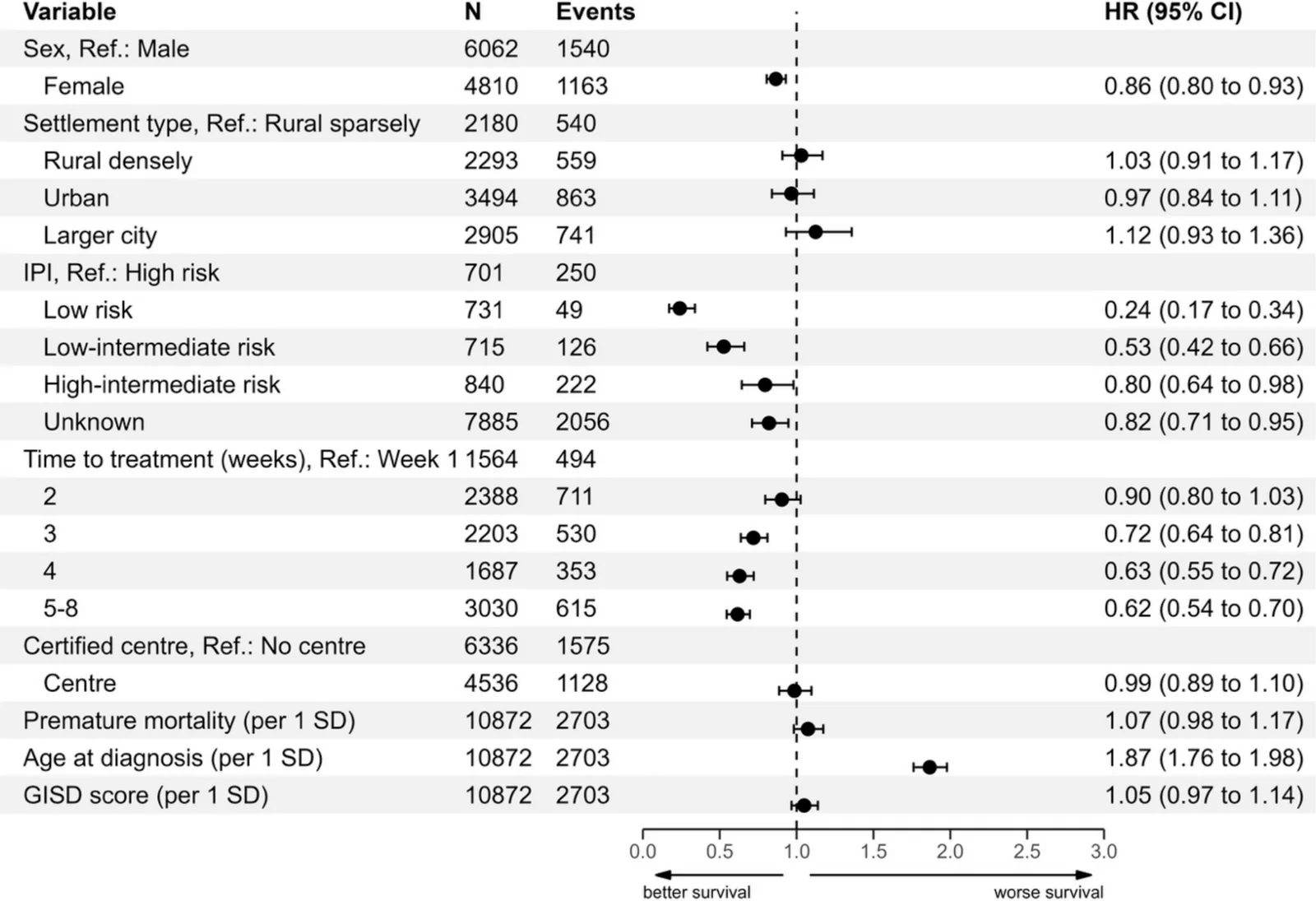
Multivariate Cox regression analysis of three-year overall survival in patients with DLBCL (95% confidence intervals). Unknown IPI score coded as a separate category

Adjustment for confounders confirmed the gradient between survival and DTI (Fig. 3; *N* = 10,872; concordance = 0.68). Compared with therapy initiation in week 1 as reference, initiation in week 2 (Hazard Ratio (HR) 0.90, 95% CI 0.80–1.03, *p* = 0.09), week 3 (HR 0.72, 95% CI 0.64–0.81, *p* < 0.001), week 4 (HR 0.63, 95% CI 0.55–0.72, *p* < 0.001), and weeks 5–8 (HR 0.62, 95% CI 0.54–0.70, *p* < 0.001) was associated with progressively lower hazards of death. Likewise, a clear gradient between survival and IPI risk was found where lower risk was associated with substantially better survival compared to the highest risk group (low risk HR 0.24, 95% CI 0.17–0.34, *p* < 0.001; low-intermediate risk HR 0.53, 95% CI 0.42–0.66, *p* < 0.001; high-intermediate HR 0.80, 95% CI 0.64–0.98, *p* = 0.01) Patients with unknown IPI had a survival most similar to those with high-intermediate IPI (HR 0.82, 95% CI 0.71–0.95, *p* = 0.004). Older age at diagnosis was associated with inferior survival (HR per standard deviation 1.87, 95% CI 1.76–1.98, *p* < 0.001), whereas female sex was associated with superior survival (HR 0.86, 95% CI 0.80–0.93, *p* < 0.001).

## Discussion

In this recent, large nationwide cohort of newly diagnosed DLBCL patients, earlier treatment with systemic therapy was associated with inferior long-term survival. Furthermore, we confirmed an expected gradient in survival across IPI risk levels, with lower risk groups being associated with significantly better outcomes than the highest-risk group. Since the timing of treatment initiation was also associated with IPI risk groups, with early treatment being observed in rather high-risk patients, the association between earlier treatment initiation and poorer survival appears to be largely driven by adverse disease characteristics rather than treatment timing itself. The univariate analysis indicated a treatment delay among patients living in districts with higher socioeconomic deprivation, which however, was not visible in the adjusted model.

Our results are in line with previous results reported by Maurer et al. [13]. In 2018, Maurer et al. demonstrated that a shorter DTI was strongly associated with adverse clinical factors, including a high IPI. However, after adjustment for IPI, the association of longer DTI with improved 2-year event-free survival remained robust [13]. These results were subsequently confirmed in a single-center real-world analysis by Camus et al. and a registry-based study by Blunt et al. published in 2019 and 2021, respectively [6, 21]. Blunt and colleagues observed a clinically significant impact of DTI in a U.S. population-based cohort, in which a shorter interval between DLBCL diagnosis and treatment initiation was associated with significantly inferior survival. The authors hypothesized that a short DTI may act as a surrogate marker for more aggressive disease biology requiring urgent therapy, although information on patients’ IPI was not available in their analysis [21]. Another large study using national cancer registry data from the U.S. investigated aggressive B-cell lymphoma in general, including a subset of DLBCL patients, reported that patients who started chemotherapy within 7 days of diagnosis had more often high IPI or advanced-stage disease. Again, after adjusting for IPI, time to treatment remained significantly associated with shorter OS [7]. However, Nikonova et al. reported results of a single-center retrospective analysis reporting that diagnostic- or treatment-related delays did not impact the survival of DLBCL patients [12]. Similarly, Lee et al. did not observe a significant impact of diagnostic wait time (DWT) defined as the interval from the initial hospital visit to diagnosis, in their single-centre retrospective analysis regarding the whole study population. However, in patients with IPI ≥ 3, longer DWT was associated with unfavourable survival [8]. Interestingly, Hay et al. observed that DTI > 8 weeks is associated with inferior OS. However, the authors also reported that lymphoma-related deaths were more frequent in patients with DTI < 4 weeks, while deaths unrelated to lymphoma or treatment were more frequent in patients with DTI > 8 weeks [11]. Based on the reviewed literature, we assume that although early treatment initiation is generally associated with poor prognosis in DLBCL, excessively prolonged delays in treatment initiation (> 8 weeks) may likewise unfavourably affect outcomes. However, the underlying mechanisms are likely different. Prolonged treatment delays > 8 weeks may be related to factors such as healthcare access, diagnostic complexity, comorbidities, or delays in referral and treatment planning [11].

Secondly, we observed that delayed treatment initiation was associated with higher district-specific socioeconomic deprivation in the univariate analysis but was not fully supported by the multivariate analysis of DTI and did not translate to a markedly higher mortality. Yet, Joonas et al. recently discovered hints of a delayed initiation of systemic therapy in DLBCL patients from Finland, depending on social determinants of health [22]. Thus, we assume that our district-level proxy for SES may not have been sensitive enough to detect larger underlying differences at the patient level. This might be a worthwhile hypothesis for identifying additional factors that mitigate SES effects on survival in DLCBL. In the narrow study cohort of DLBCL patients analyzed here, treated within 56 days after diagnosis, neither premature mortality as a proxy for comorbidity burden nor socioeconomic deprivation affected survival, whereas our earlier research in unselected DLBCL patients underlined the importance of both factors [15]. Combining our previous findings on how SES impacts DLBCL prognosis with the current study on DTI’s influence, we assume that the higher mortality observed in DLBCL patients from socioeconomically deprived regions is unlikely to be primarily driven by delayed treatment initiation. Instead, it may result from variations in treatment adherence or intensity, e.g., due to a higher comorbidity burden among patients living in higher socioeconomically deprived areas.

Although we report results from a large and up-to-date cohort of DLBCL patients derived from the national cancer registry, several important limitations should be noted. Data on IPI were available for less than 30% of patients analyzed. To overcome this reporting bias, we performed sensitivity analyses restricted to patients with available IPI data and additionally performed imputation. These analyses were confirmative to the main findings in terms of timing of treatment initiation and in line with earlier work. Moreover, the cancer registry data did not include information on single-substance dosing. In newly diagnosed DLBCL, reduced relative dose intensity of anthracycline-containing immunochemotherapy has been associated with inferior outcomes, as reported by several retrospective studies, irrespective of age groups [23]. In addition, other common biological prognostic factors, such as cell of origin or presence of bulky disease, were not available in the cancer registry dataset. Furthermore, individual-level clinical variables including disease stage, ECOG performance status, lactate dehydrogenase levels, and comorbidity indices were either unavailable or incompletely recorded in the cancer registry dataset, representing important unmeasured confounders that may have influenced the observed associations between treatment timing and survival. Furthermore, the exclusion of patients with a follow-up of less than 56 days may have introduced a selection bias, as patients who died very shortly after diagnosis were systematically excluded. Consequently, the results may not be generalizable to the most severely ill patients and may underestimate mortality in high-risk groups. Moreover, the group without a report on systemic therapy initiation cannot be further characterized with the available data, as it remains unclear whether these patients truly received no systemic treatment or whether treatment was administered but not captured in the registry due to reporting gaps. This informative missingness limits the interpretation of this subgroup and may affect the generalizability of the findings. Nevertheless, in future analyses, we aim to investigate the impact of additional non-biological determinants by integrating complementary data sources, including health insurance claims data and data from the German national cohort [24].

Our findings confirm the previous assumption that early treatment initiation in DLBCL is primarily driven by adverse disease characteristics, with high-risk patients requiring more urgent therapy.

However, a residual association between longer DTI and improved survival persisted after adjustment for IPI, suggesting that treatment timing may represent an independent prognostic factor, particularly in patients with less aggressive disease.

## Supplementary Information

Supplementary Material 1.

## Data Availability

The data included in the study were analyzed in the protected environment of the Hamburg Cancer Registry. Due to data protection and security regulations, the sensitive patient data cannot be made publicly available. In general, however, data for scientific projects can be requested from the Hamburg Cancer Registry. All analysis scripts can be made available on request from the corresponding author.
