# Supplementary material for: Insights into the survival impact of delayed immunochemotherapy in newly diagnosed diffuse large B-cell lymphoma: results from a nationwide registry study
Source: BMC Cancer. 2026 Sep 26;26:1144. doi: 10.1186/s12885-026-17024-9 (PMC13615582; doi:10.1186/s12885-026-17024-9)
Supplement: Supplementary file 1 [file 12885_2026_17024_MOESM1_ESM.docx]

**Supplementary Methods S1: Study Design and Data Sources**

We conducted a retrospective population-based cohort study using a validated and harmonised pooled clinical cancer registry dataset, mandated by federal law since 2013, provided by the Centre for Cancer Registry Data at the Robert Koch Institute, covering the German population (approximately 83 million inhabitants) for the years 2020–2023, with vital status follow-up until 31 December 2024. Further details on the mandatory clinical cancer registration in Germany are described elsewhere (14). The feasibility and validity of German cancer registry data to assess DLBCL outcomes was demonstrated in earlier publications (15–17).

The data from the cancer registry is available in different regional resolutions: the 16 federal states at the upper level, corresponding to the Nomenclature des Unités territoriales statistiques (NUTS) hierarchical level 1, and 401 regional districts (territorial status of the year 2020), corresponding to the NUTS-3 level. Furthermore, we extracted aggregated data on the degree of urbanity and premature mortality at the district level (NUTS-3) from the ongoing spatial observation of the Federal Institute for Research on Building, Urban Affairs, and Spatial Development (18). Data on regional deprivation at the NUTS-3 level per calendar year are based on the German Index of Socioeconomic Deprivation (GISD) of the Robert Koch Institute (19). Information on the location of certified centers per ZIP code was extracted from the database of the German Cancer Society (Deutsche Krebsgesellschaft - DKG), online available at [www.oncomap.de](http://www.oncomap.de) (data accessed at 01/04/2026. The term certified centers includes certification by the DKG, European Cancer Center, or by ÄKzert® - Zertifizierungsstelle der Ärztekammer Westfalen-Lippe). Finally, data on age- and sex-specific one-year death probabilities for the general population based on life tables at the level of Germany were extracted from the Human Mortality Database of the Max Planck Institute for Demographic Research, University of California, Berkeley, and French Institute for Demographic Studies (20).

**Supplementary Methods S2: Sensitivity analysis**

As sensitivity analysis of the central survival Cox proportional hazards model (model II) with respect to missingness for the IPI, we estimated a complete-case analysis including only patients with known IPI on one hand, and multiple imputation of missing IPI values by chained equations (20 iterations) using the first imputed dataset on the other hand. We did not apply adjustments for multiple comparisons or report p-values, as the analyses were based on population-level data rather than estimates subject to sampling variability. Moreover, the study was not designed for formal hypothesis testing but was conducted as an exploratory analysis. For reporting our results, we adhered to the Strengthening the Reporting of Observational Studies in Epidemiology (STROBE) statement (21).

**Table S1.** Baseline characteristics of the study cohort stratified comparing patients with treatment initiation within eight weeks to those after eight weeks and patients without report on treatment

| **Variables** | **Within 8 weeks (N=10,872)** | **After 8 weeks (N=1,063)** | **No report on treatment (N=4,508)** | **SMD** |
| --- | --- | --- | --- | --- |
| **Year of diagnosis, n (%)** |  |  |  | 0.06 |
| 2020 | 2,906 (26.7) | 273 (25.7) | 1,106 (24.5) |  |
| 2021 | 2,909 (26.8) | 262 (24.6) | 1,190 (26.4) |  |
| 2022 | 2,762 (25.4) | 284 (26.7) | 1,202 (26.7) |  |
| 2023 | 2,295 (21.1) | 244 (23.0) | 1,010 (22.4) |  |
| **Age in years, median (IQR)** | 71 (61, 80) | 72 (62, 79) | 75 (64, 83) | 0.16 |
| **Age group, n (%)** |  |  |  | 0.21 |
| 18–39 | 469 (4.3) | 34 (3.2) | 145 (3.2) |  |
| 40–49 | 571 (5.3) | 48 (4.5) | 179 (4.0) |  |
| 50–59 | 1,353 (12.4) | 138 (13.0) | 446 (9.9) |  |
| 60–69 | 2,467 (22.7) | 256 (24.1) | 845 (18.7) |  |
| 70–79 | 3,160 (29.1) | 327 (30.8) | 1,218 (27.0) |  |
| 80+ | 2,852 (26.2) | 260 (24.5) | 1,675 (37.2) |  |
| **Female sex, n (%)** | 4,810 (44.2) | 471 (44.3) | 2,046 (45.4) | 0.02 |
| **IPI, n (%)** |  |  |  | 0.43 |
| IPI H (High risk) | 701 (6.4) | 36 (3.4) | 59 (1.3) |  |
| IPI L (Low risk) | 731 (6.7) | 53 (5.0) | 58 (1.3) |  |
| IPI L/I (Low-intermediate risk) | 715 (6.6) | 58 (5.5) | 55 (1.2) |  |
| IPI H/I (High-intermediate risk) | 840 (7.7) | 37 (3.5) | 66 (1.5) |  |
| IPI unknown | 7,885 (72.5) | 879 (82.7) | 4,270 (94.7) |  |
| **Deaths within three years, n (%)** | 2,703 (24.9) | 251 (23.6) | 1,285 (28.5) | 0.07 |
| **Spatial type, n (%)** |  |  |  | 0.10 |
| Rural sparsely | 2,180 (20.1) | 203 (19.1) | 933 (20.7) |  |
| Rural densely | 2,293 (21.1) | 237 (22.3) | 962 (21.3) |  |
| Urban | 3,494 (32.1) | 356 (33.5) | 1,652 (36.6) |  |
| Larger city | 2,905 (26.7) | 267 (25.1) | 961 (21.3) |  |
| **Certified hospital center, n (%)** | 4,536 (41.7) | 410 (38.6) | 1,502 (33.3) | 0.12 |
| **Socioeconomic status, n (%)** |  |  |  | 0.07 |
| High | 2,649 (24.4) | 236 (22.2) | 1,140 (25.3) |  |
| Middle | 6,520 (60.0) | 666 (62.7) | 2,779 (61.6) |  |
| Low | 1,703 (15.7) | 161 (15.1) | 589 (13.1) |  |
| **GISD score, median (IQR)** | 0.50 [0.39, 0.62] | 0.50 [0.40, 0.62] | 0.50 [0.38, 0.60] | 0.05 |
| **District level deaths before age 70, per 1,000 persons, median (Q1, Q3)** | 2.86 (2.51, 3.63) | 2.87 (2.51, 3.53) | 2.86 (2.50, 3.39) | 0.09 |

Abbreviations: IPI: International Prognostic Index; IQR: Interquartile range; Q1: First quartile; Q3: Third quartile; SD: Standard deviation; SMD: Standardized Mean Difference. Values ≥0.10 were interpreted as non-negligible differences.

**Figure S1.** Multivariate linear regression analysis of treatment initiation within eight weeks in patients with DLBCL (95% confidence intervals).

**Figure S2.** Multivariate Cox regression analysis of three-year overall survival in patients with DLBCL (95% confidence intervals). Complete case analysis excluding patients with unknown IPI (concordance = 0.72).

**Figure S3.** Multivariate Cox regression analysis of three-year overall survival in patients with DLBCL (95% confidence intervals). Multiple imputation applied for unknown IPI score (concordance = 0.70)

**Supplementary Results S1: Sensitivity analyses**

In the complete-case analysis 2,987 patients remained with complete IPI reporting (**Figure S2,** concordance 0.72) and a marked reduction in mortality risk was only visible for those with treatment initiation in week 4 (HR 0.65, 95% CI 0.5-0.83) and in week 5-8 (HR 0.74, 95% CI 0.56-0.98) relative to week 1. In contrast, the model with imputed IPI (**Figure S3**, concordance 0.70) yielded estimates more closely aligned with the primary analysis (week 3: HR 0.73, 95% CI 0.65-0.82; week 4: HR 0.63, 95% CI 0.55-0.72; weeks 5-8: HR 0.63, 95% CI 0.56-0.71).
